# Connexin diversity in the heart: insights from transgenic mouse models

**DOI:** 10.3389/fphar.2013.00081

**Published:** 2013-06-27

**Authors:** Sander Verheule, Sven Kaese

**Affiliations:** ^1^Department of Physiology, Faculty of Medicine, Maastricht UniversityMaastricht, Netherlands; ^2^Department of Cardiovascular Medicine, Division of Electrophysiology, University of MuensterMuenster, Germany

**Keywords:** gap junctions, connexins, mouse models, conduction, arrhythmias, cardiac

## Abstract

Cardiac conduction is mediated by gap junction channels that are formed by connexin (Cx) protein subunits. The connexin family of proteins consists of more than 20 members varying in their biophysical properties and ability to combine with other connexins into heteromeric gap junction channels. The mammalian heart shows regional differences both in connexin expression profile and in degree of electrical coupling. The latter reflects functional requirements for conduction velocity which needs to be low in the sinoatrial and atrioventricular nodes and high in the ventricular conduction system. Over the past 20 years knowledge of the biology of gap junction channels and their role in the genesis of cardiac arrhythmias has increased enormously. This review focuses on the insights gained from transgenic mouse models. The mouse heart expresses Cx30, 30.2, 37, 40, 43, 45, and 46. For these connexins a variety of knock-outs, heart-specific knock-outs, conditional knock-outs, double knock-outs, knock-ins and overexpressors has been studied. We discuss the cardiac phenotype in these models and compare Cx expression between mice and men. Mouse models have enhanced our understanding of (patho)-physiological implications of Cx diversity in the heart. In principle connexin-specific modulation of electrical coupling in the heart represents an interesting treatment strategy for cardiac arrhythmias and conduction disorders.

## Introduction

**Figure 1:**
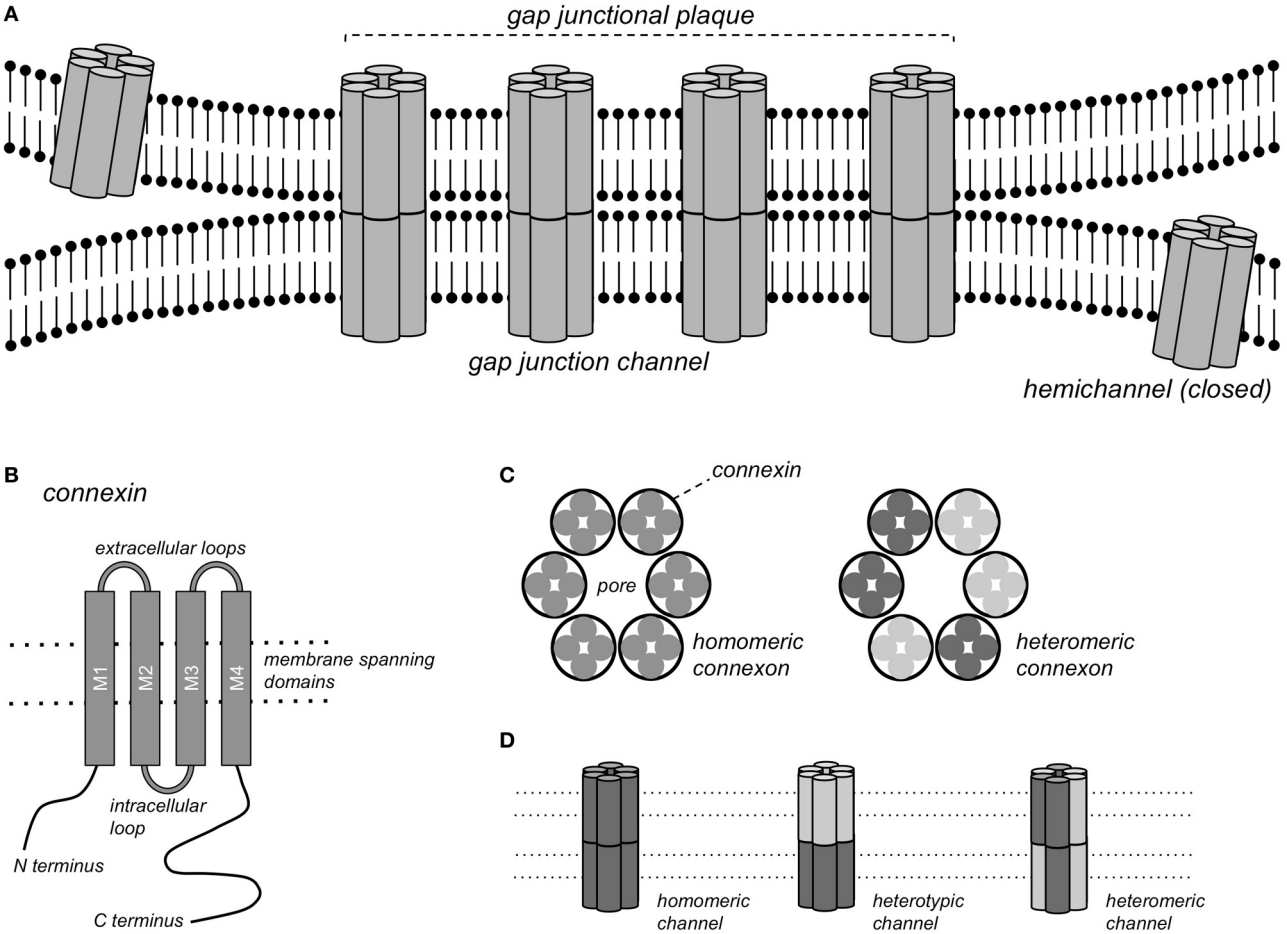
**Gap junction channels. (A)** Schematic representation of a gap junctional plaque, with each of the neighboring cells contributing one hemichannel to form the complete gap junction channel. **(B)** Structure of the pattern of a single connexin subunit with 4 domains traversing the membrane. **(C)** A hemichannel or connexon consists of a hexagonal arrangement of 6 Cx subunits, either of the same Cx isoform (homomeric connexon) or of different isoforms (heteromeric connexon). **(D)** Gap junction channels formed by homomeric connexons of the same isoform (homomeric channel), by homomeric connexons of different isoforms (heterotypic channel) or by heteromeric connexons (heteromeric channels).

Gap junction channels form continuous pores between the cytoplasms of closely apposed cells that are permeable to ions and molecules <1 kDa, thereby conferring electrical and metabolic coupling between neighboring cells. Gap junction channels consist of connexin (Cx) protein subunits. More than 20 Cx isoforms have been described in both humans and mice and mutations in Cx genes have been implicated in various inheritable diseases (Sohl and Willecke, 2004; Dobrowolski and Willecke, 2009). Complete gap junction channels are formed by the docking of two hemichannels, or connexons, contributed by closely apposed cells (Figure 1). Each connexon consists of 6 connexin subunits, and a homomeric gap junction channel consists of 12 Cx proteins of the same isoform. When two homomeric connexons consisting of different connexins are apposed, a heterotypic channel may form if those connexins are compatible. In heteromeric connexons, different isoforms are mixed, and a heteromeric channel may form. Some Cxs also form functional hemichannels, i.e., connexons that can open under certain conditions, causing depolarization of the plasma membrane (John et al., 1999; Bukauskas et al., 2006).

Different regions of the heart show specific profiles of Cx expression, which in mouse and human hearts show many similarities, but also some important differences (Table 1). Different parts of the heart also have varying requirements for the degree of electrical coupling. To spread the activation wave rapidly over the ventricles, the large Purkinje myocytes of the specialized ventricular conduction system need to be strongly coupled. To create a delay between the atrial and ventricular contraction, a very low degree of coupling is required in the atrioventricular (AV) node. Similarly, to allow pacemaker function, pacemaker myocytes in the sinoatrial (SA) node need to be weakly coupled, otherwise the pacemaker (or ectopic focus) would in effect be silenced by the surrounding working myocardium (Joyner and van Capelle, 1986; Joyner et al., 2000).

**Table 1:** **Comparison of Cx regional expression in the mouse and human heart**.

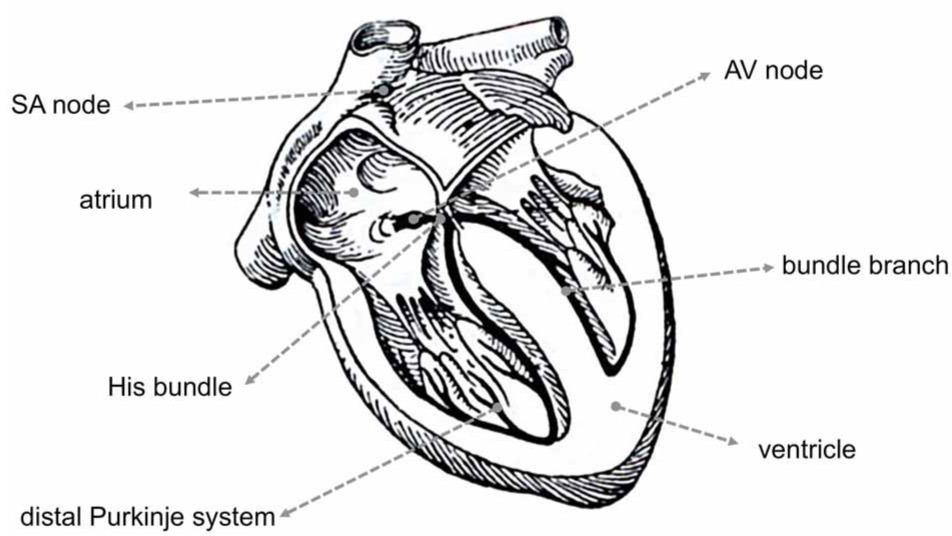				
	**Mouse**	**References**	**Human**	**References**
SA node	Cx45, Cx30.2, (Cx30, Cx46)	Verheijck et al., 2001; Kreuzberg et al., 2005; Chi et al., 2010; Gros et al., 2010	Cx45, Cx40	Davis et al., 1995; Chandler et al., 2009; Kreuzberg et al., 2009
Atria	Cx40, Cx43, ((Cx45))	Delorme et al., 1995, 1997; Alcoléa et al., 2004; Miquerol et al., 2004	Cx40, Cx43, (Cx45)	Davis et al., 1995; Vozzi et al., 1999; Kanagaratnam et al., 2002; Kreuzberg et al., 2009; Greener et al., 2011
AV node (compact)	Cx45, Cx30.2	Coppen et al., 2001; Miquerol et al., 2004; Kreuzberg et al., 2006	Cx40, Cx45	Davis et al., 1995; Kreuzberg et al., 2009; Greener et al., 2011
His bundle	Cx40, Cx45, (Cx30.2, Cx46)	Simon et al., 1998; Coppen et al., 1999, 2001; Kreuzberg et al., 2006; Chi et al., 2010	Cx40, Cx43	Hucker et al., 2008; Greener et al., 2011
Bundle branches	Cx40, Cx45, (Cx46)	Simon et al., 1998; Coppen et al., 1999, 2001; van Rijen et al., 2001; Chi et al., 2010	Cx45	Davis et al., 1995; Coppen et al., 2003; Kreuzberg et al., 2009; Greener et al., 2011
Distal Purkinje system	Cx40, Cx43, Cx45, (Cx46)	Coppen et al., 1999; Miquerol et al., 2003; Chi et al., 2010	Cx40, Cx43, (Cx45)	van Kempen et al., 1995; Coppen et al., 2003; Kreuzberg et al., 2009
Ventricles	Cx43, ((Cx45))	Delorme et al., 1997; Stein et al., 2009; Bao et al., 2011	Cx43, (Cx45)	Davis et al., 1995; Vozzi et al., 1999; Dupont et al., 2001; Kreuzberg et al., 2009; Greener et al., 2011

(Cx) and ((Cx)) signify relatively small and trace amounts.

Cardiac gap junctions are highly dynamic structures, with a Cx43 protein half-life of a few hours and a comparably rapid redistribution in response to e.g., ischemia (Beardslee et al., 1998; Smyth et al., 2012). Connexins interact directly with numerous other cellular proteins, e.g., cytoskeletal components (Herve et al., 2007). Apart from their well-established roles in electrical and metabolic coupling, a number of other functions of Cx proteins are emerging, for example in mechanical adhesion and interactions with voltage-gated membrane channels (Agullo-Pascual and Delmar, 2012) and, in the case of Cx43, in mitochondrial metabolism (Ruiz-Meana et al., 2008). Conversely, electrical coupling may be mediated not only by gap junction channels, but also to some degree by ephaptic coupling, i.e., by electric field effects (Sperelakis, 2002; Lin and Keener, 2010).

Connexins differ in various biophysical properties, such as single channel conductance, permeability to larger (dye) molecules and sensitivity to the cytoplasmic pH and transjunctional potential difference, (see e.g., Elfgang et al., 1995; Gonzalez et al., 2007; Rackauskas et al., 2010). Concerning the latter, gap junction channels may close during prolonged, large voltage differences between cells, for example when one myocyte fires an action potential while its direct neighbor remains at rest (Lin and Veenstra, 2004; Lin et al., 2005). While such large voltage gradients can be imposed in voltage clamp experiments, they are unlikely to occur in the heart. Even in regions with a relatively low degree of electrical coupling, such as the SA node, transjunctional potential differences >10 mV last only a few milliseconds (Verheule et al., 2001), which would be too short for the slow process of voltage-dependent inactivation of gap junction channels.

Cx trafficking, degradation, channel gating and permeability can be regulated by phosphorylation of serine and tyrosine residues (Solan and Lampe, 2009). During ischemia, intracellular acidification, release of lipids and channel phosphorylation cause channel closure and redistribution (Dhein, 2006). This response to ischemia allows the confinement of diseased from healthy myocardium, thus enabling the myocardium to “heal-over” (de Mello et al., 1969). On the other hand, redistribution of connexins under pathological conditions has been implicated in arrhythmogenesis (Salameh and Dhein, 2011; Duffy, 2012).

Much of our current knowledge of cardiac connexins stems from studies on mouse models (Table 2). There are numerous similarities, but also some important differences between the hearts of mice and humans (Kaese and Verheule, 2012). In this review, we will discuss evidence from genetically engineered mouse models on the properties and roles of the various cardiac connexins, highlighting both their role during cardiac development and in the adult heart.

**Table 2:** **Overview of genetically engineered mouse models**.

**Type**	**Model**	**Initial description**
Knock-out	Cx30^−/−^	Gros et al., 2010
	Cx30.2^−/−^	Kreuzberg et al., 2006
	Cx37^−/−^	Simon et al., 1997; Munger et al., 2013
	Cx40^−/−^	Kirchhoff et al., 1998; Simon et al., 1998
	Cx43^−/−^	Reaume et al., 1995
	Cx45^−/−^	Kumai et al., 2000
	Cx46^−/−^	Chi et al., 2010
Conditional Knockout	Cx43 inducible	Eckardt et al., 2004
	Cx43 heart-specific	Gutstein et al., 2001a
	Cx43 chimera	Gutstein et al., 2001b
	Cx45 heart-specific	Frank et al., 2012
Overexpression	Cx43	Ewart et al., 1997
	Cx45	Betsuyaku et al., 2006
Double knock-outs	Cx30.2^−/−^–Cx40^−/−^	Schrickel et al., 2009
	Cx30.2^−/−^–Cx45^inducible^	Frank et al., 2012
	Cx32^−/−^–Cx43^−/−^	Houghton et al., 1999
	Cx37^−/−^–Cx40^−/−^	Simon et al., 2004
	Cx40^−/−^–Cx43^−/−^ and ^+/−^	Kirchhoff et al., 2000; Simon et al., 2004
	Cx40^−/−^–Cx45^+/−^	Kruger et al., 2006
Knock-ins	Cx40 KI Cx45	Alcoléa et al., 2004
	Cx43 KI Cx26	Winterhager et al., 2007
	Cx43 KI Cx31	Zheng-Fischhofer et al., 2006
	Cx43 KI Cx32	Plum et al., 2000
	Cx43 KI Cx40	Plum et al., 2000
	Cx45 KI Cx36	Frank et al., 2010
Mutants	Cx43 K258stop	Maass et al., 2004, 2007
	Cx43 S325/328/330 E or A	Remo et al., 2011
	Cx43 I130T	Kalcheva et al., 2007
	Cx43 G138R	Dobrowolski et al., 2008
	Cx43 G60S	Flenniken et al., 2005; Manias et al., 2008

## Connexin 30

Cx30 forms channels with a large single channel conductance (γ*j*) of 179 pS (in K-aspartate), a potential of half-maximal inactivation (V_1/2_) of 27 mV and a voltage-independent fraction (*G*_*j*, min_) of 0.15 (Valiunas et al., 1999). Cx30 is expressed at low abundance in the murine SA node (Gros et al., 2010). Interestingly, Cx30-deficient mice have a 9% higher heart rate than wild type (Wt) mice and a lower beat-to-beat variability (Gros et al., 2010). This increased rate was still present under autonomic blockade, indicating that Cx30 affects the intrinsic pacemaker frequency. The presence of Cx30 could conceivably enhance electrical coupling between the SA node and the surrounding atrium and thereby decelerate impulse formation within the SA node (Dhein, 2010; Gros et al., 2010). However, it is not known whether Cx30 in the SA node forms heteromeric gap junction channels with the other connexins present (Cx30.2 and Cx45), and whether this would increase or decrease cell-to-cell electrical coupling.

## Connexin 30.2

Cx30.2, the murine ortholog of human Cx31.9 (Belluardo et al., 2001), forms small conductance, weakly voltage-dependent channels [γ*j* = 9 pS, V_1/2_ > 60 mV (Kreuzberg et al., 2005)]. Cx30.2 is expressed in the murine SA node, AV node and His bundle (Kreuzberg et al., 2005). In transfected HeLa cells, Cx30.2 can form both heterotypic (Kreuzberg et al., 2005) and heteromeric (Gemel et al., 2008) channels with Cx40, Cx43, and Cx45, the major myocardial connexins. Cx30.2 was colocalized with Cx45 in the SA and AV nodes, but not with Cx40 and Cx43 (Kreuzberg et al., 2005). Surprisingly, Cx30.2 deficient mice display accelerated AV nodal conduction, a decrease in AV Wenckebach period and a higher ventricular rate during atrial fibrillation (Kreuzberg et al., 2006). In addition, whereas deletion of Cx40 slows AV conduction, it is normal in mice deficient for both Cx30.2 and Cx40 (Schrickel et al., 2009), suggesting that the balance between Cx40 and Cx30.2 is an important determinant of AV conduction in mice. Cx30.2 is able to form functional hemichannels (Bukauskas et al., 2006), which in the open state would decrease the membrane resistance and thereby slow conduction, but the role of these hemichannels in the adult heart is uncertain at present. During development, Cx30.2 expression and proper development of the AV conduction system is determined by an enhancer region under the control of Tbx5 and GATA4. Accordingly, the PR interval is shortened in GATA4^+/−^ mice (Munshi et al., 2009). Moreover, inhibition of Notch signaling during development leads to a loss of Cx30.2 expressing cells, hypoplasia of the AV node and accelerated AV conduction (Rentschler et al., 2011). It is important to note that Cx31.9, the human ortholog of murine Cx30.2, is not expressed in the human SA and AV nodes, ventricular conduction system or working myocardium (Kreuzberg et al., 2009). This indicates that the presence of Cx30.2 may reflect an adaptation in the small mouse heart to allow high activation frequencies and optimize A-V timing. In addition to this difference, AV conduction system in the mouse is also electrically connected to the ventricles at the basal part of the septum, leading to a baso-apical activation pattern within the septum (van Rijen et al., 2001).

## Connexin 37

Cx37 forms large conductance channels that are moderately voltage sensitive [γ*j* = 200–250 pS in K-glutamate, V_1/2_ = 28 mV, *G*_*j*, min_ = 0.27 (Reed et al., 1993)]. In the adult heart, Cx37 is expressed by endothelial cells in blood vessels and the endocardial lining of the chambers, although expression has also been observed in parts of the ventricular myocardium during embryonic development (Haefliger et al., 2000). Cx37^−/−^ mice do not develop venous and lymphatic valves (Munger et al., 2013). In addition, mice lacking both Cx37 and Cx40 show a high incidence of atrial and ventricular septal defects at birth (Simon et al., 2004).

## Connexin 40

### Properties and expression

Cx40 forms channels with a large single channel conductance and moderate voltage-sensitivity [γ*j* = 150 pS in KCl, V_1/2_ = 44 mV, *G*_*j*, min_ = 0.5 (Traub et al., 1994)]. In the developing mouse heart, Cx40 is widely expressed in the ventricles and atria at embryonic day 11. From day 14 onwards, Cx40 becomes restricted to the conduction system in the ventricles, but it remains present in the atrial working myocardium (Delorme et al., 1995). The transcription factors Tbx2 and Tbx3 repress Cx40 expression (Hoogaars et al., 2004; Aanhaanen et al., 2011). Inactivation of Tbx2 leads to the formation of Cx40-expressing accessory pathways and ventricular preexcitation (Aanhaanen et al., 2011). On the other hand, Cx40 expression is increased by Nkx2-5 (Harris et al., 2006) and Tbx5 (Pizard et al., 2005; Arnolds et al., 2012) that delineate the conduction system. In fact, normal development of the ventricular conduction system requires the expression of Cx40 (Sankova et al., 2012).

In the adult mouse heart, Cx40 is expressed by atrial myocytes and ventricular conduction system (His bundle, left and right bundle branches and Purkinje network) (Simon et al., 1998; Miquerol et al., 2004; van Veen et al., 2005b). The selective expression of Cx40 by the conduction system within the ventricle has allowed sophisticated functional studies on conduction within the Purkinje network (Miquerol et al., 2004; Tallini et al., 2007).

### Atria

Information on the effect of Cx40-deficiency on atrial conduction is somewhat contradictory. In the first studies, the *P* wave duration was significantly prolonged (Kirchhoff et al., 1998; Simon et al., 1998; Hagendorff et al., 1999; Verheule et al., 1999; Bagwe et al., 2005). Some other studies have not reproduced this observation (Bevilacqua et al., 2000; Tamaddon et al., 2000; Vanderbrink et al., 2000). Measurement of P-wave duration in mice is not straightforward, requiring different leads to accurately determine the end of the biphasic P-wave. In two studies that measured atrial conduction directly using direct contact mapping or optical mapping, a reduction in conduction velocity was observed (Verheule et al., 1999; Bagwe et al., 2005). However, a later study used optical mapping to show that deletion of Cx40 did not affect conduction velocity, but did abolish the difference in conduction velocity between the left and right atria (Leaf et al., 2008).

Atrial myocytes express both Cx40 and 43. Based on expression studies in Xenopus oocytes, Cx40 and 43 were originally thought to be incompatible (White and Bruzzone, 1996). However, later studies presented compelling evidence that Cx40 and 43 can form both heterotypic (Valiunas et al., 2000) and heteromeric (He et al., 1999; Cottrell and Burt, 2001) gap junction channels in mammalian cells, including adult atrial myocytes (Elenes et al., 1999). In cultured neonatal mouse atrial myocytes, Cx40 and 43 appear to make equal contributions to total gap junctional conductance (Lin et al., 2010). Intriguingly, a study on cultured strands of atrial myocytes that the Cx40/Cx43 ratio was an important determinant of propagation, with Cx43 increasing and Cx40 decreasing conduction velocity (Beauchamp et al., 2006).

In intact Cx40^−/−^ mice, episodes of atrial tachyarrythmias/atrial fibrillation could be induced by transesophageal pacing (Hagendorff et al., 1999) and direct atrial pacing (Verheule et al., 1999; Bevilacqua et al., 2000). In perfused Cx40^−/−^ mouse hearts studied with optical mapping, atrial ectopic beats and intraatrial conduction block during pacing at high rates were reported (Bagwe et al., 2005).

### Sinoatrial node

In contrast to larger species, such as rabbits (Verheule et al., 2001), pacemaker myocytes in the central SA node do not express Cx40 (Verheijck et al., 2001; Wiese et al., 2009) However, Cx40-deficiency does cause a modest increase in sinus cycle length (Hagendorff et al., 1999; Verheule et al., 1999; Bevilacqua et al., 2000; de Wit et al., 2003), and an increase in (corrected) SA node recovery time (Hagendorff et al., 1999; Verheule et al., 1999). The effects of Cx40-deficiency on SA node function may be caused either by altered conduction from the SA node to the atrium and/or by the phenomenon that the “atrial pacemaking complex” may be much larger than the area that is classically considered to be the sinus node (Glukhov et al., 2010).

### Atrioventricular conduction

AV conduction is also affected in Cx40^−/−^ mice, reflected in a prolonged PR interval, increased QRS duration and a QRS morphology reminiscent of right bundle branch block in humans (Kirchhoff et al., 1998; Simon et al., 1998; Verheule et al., 1999; Bevilacqua et al., 2000; Vanderbrink et al., 2000). In addition, episodes of 2nd and 3rd degree AV block were observed in Cx40^−/−^ mice (Kirchhoff et al., 1998; Hagendorff et al., 1999). With minor differences between studies, Cx40-deficiency prolongs the AV effective refractory period (ERP), the cycle lengths of A to V Wenckebach and 2:1 block, while the atrial ERP, ventricular ERP, cycle lengths of V to A Wenckebach and 2:1 block are not affected (Hagendorff et al., 1999; Verheule et al., 1999; Bevilacqua et al., 2000; Vanderbrink et al., 2000). Although Cx40 is not expressed in the central AV node (Delorme et al., 1995; Simon et al., 1998; Coppen et al., 1999), the AH interval is prolonged in intact mice (Bevilacqua et al., 2000; Vanderbrink et al., 2000), AV nodal conduction curves are shifted (Vanderbrink et al., 2000) and AV nodal facilitation is reversed (Zhu et al., 2005), indicative of an effect on the AV node itself. However, AV nodal conduction curves recorded in perfused hearts did not differ between Wt and Cx40^−/−^ mice (van Rijen et al., 2001), suggesting a possible role of autonomic activity in the observations in intact mice.

Both the left and right bundle branch normally express high levels of Cx40 (Simon et al., 1998; van Rijen et al., 2001). High resolution mapping has revealed that deletion of Cx40 leads to slower conduction in the left bundle branch and conduction block in the thinner right bundle branch, causing a delayed activation of the right ventricle (Tamaddon et al., 2000; van Rijen et al., 2001). Interestingly, in mice the common bundle (expressing Cx40 and 45) is electrically connected to the base of the interventricular septum (expressing Cx43) through a small transitional zone expressing Cx43 and 45 (van Rijen et al., 2001; van Veen et al., 2005b) This arrangement results in an activation pattern within the septum from base to apex that is quite different from the pattern in humans (Durrer et al., 1970).

### Ventricles

In the ventricular myocardium, which expresses Cx43 but not Cx40, conduction velocity is unaffected by Cx40-deficiency and the inducibility of ventricular arrhythmias under normal conditions was not increased (Verheule et al., 1999; Bevilacqua et al., 2000; Tamaddon et al., 2000), although one study reported an increased inducibility of ventricular tachycardia (VT) in Cx40^−/−^ mice during infusion of isoproterenol (Bevilacqua et al., 2000), possibly involving the Purkinje system.

In none of the studies mentioned above did heterozygous Cx40^+/−^ mice display a electrophysiological phenotype that differed from Cx40^+/+^ mice, indicating that even a substantial reduction in Cx40 protein does not greatly affect cardiac conduction in mice. Moreover, although it differs considerably in its biophysical properties, Cx45 can replace Cx40 almost completely, because cardiac activation in Cx40KICx45 mice is normal, except for a slower conduction velocity in the left atrium and right bundle branch (Alcoléa et al., 2004).

Two factors complicate the interpretation of electrophysiological data from Cx40^−/−^ mice. First, although this was not noted in the earliest studies, Cx40 deficient mice display a high incidence of a variety of cardiac malformations, including ventricular septal defects, tetralogy of Fallot, double-outlet right ventricles, endocardial cushion and aortic arch defects (Kirchhoff et al., 2000; Gu et al., 2003). The occurrence of cardiac malformations is exacerbated in mice in which a homozygous deletion of Cx40 is combined with a heterozygous deletion of either Cx43 (Kirchhoff et al., 2000) or Cx45 (Kruger et al., 2006), leading to neonatal lethality in the former and additional atrial defects and further delayed AV conduction in the latter. Mice that are homozygously deficient for both Cx40 and 43 die much earlier than Cx43^−/−^ mice, around embryonic day 12, with an abnormal rotation of the ventricles (Simon et al., 2004). Second, apart from expression by atrial myocytes and Purkinje cells, Cx40 is also expressed by endothelial cells (Dahl et al., 1995), together with Cx37. Interestingly, de Wit et al have reported that in Cx40^−/−^ mice, arterioles show spontaneous, irregular vasomotion and that blood pressure is greatly increased from 90 to 120 mmHg (de Wit et al., 2003). To what extent hypertension in Cx40^−/−^ mice is responsible for secondary changes in cardiac pump function, autonomic regulation and electrophysiology (e.g., decreased heart rate and slower AV nodal conduction) is uncertain at present.

## Connexin 43

### Properties and expression

Cx43 forms channels with a single channel conductance of 120 pS (in CsCl) and a modest voltage dependence (V_1/2_ = 60 mV and *G*_*j*, min_ = 0.4) (Elenes et al., 2001).

In the developing mouse heart, Cx43 is specifically present in the trabeculated parts of the ventricle at embryonic day 12.5, but is abundantly expressed by the entire working myocardium later on (Coppen et al., 2003; Miquerol et al., 2003). Cx43 expression is suppressed by the transcription factors Tbx18 (specifying the SA node), Tbx2 and Tbx3 (specifying the AV conduction system) (Christoffels et al., 2004; Bakker et al., 2008; Kapoor et al., 2011; Sizarov et al., 2011).

Cx43^−/−^ mice die shortly after birth from respiratory failure caused by a right ventricular outflow tract obstruction (Reaume et al., 1995). Abnormal cardiac development is already visible during the looping phase at embryonic day 10 (Ya et al., 1998). The outflow tract defect can be traced back to the neural crest, where abnormal p53 activation in Cx43^−/−^ mice causes apoptosis of primordial germ cells that would otherwise have migrated to the heart (Lo et al., 1997; Francis and Lo, 2006). Both a deficiency and an excess of Cx43 can derail this process (Ewart et al., 1997; Huang et al., 1998), although it does not necessarily depend on Cx43 expression in the neural crest itself (Kretz et al., 2006). In addition to this defect, Cx43^−/−^ (and Cx43^+/−^) mice also show abnormal patterning of the main coronary arteries (Li et al., 2002; Walker et al., 2005; Liu et al., 2006).

### Ventricular conduction

Using optical mapping in Cx43^−/−^ mouse embryos, Vaidya et al reported that ventricular conduction velocity was unaffected at embryonic day 12.5, when Cx40 is still present abundantly in the ventricle. At embryonic day 17.5 however, ventricular conduction velocity was greatly reduced and ventricular arrhythmias were frequently observed (Vaidya et al., 2001). Accordingly, gap junctional coupling and conduction velocity are dramatically reduced in cultured neonatal myocytes from Cx43^−/−^ mice, which do not show a compensatory increase in Cx40 and 45 (Beauchamp et al., 2004; Vink et al., 2004).

In adult mice, Cx43 is expressed in the atrial and ventricular working myocardium and in the distal Purkinje system (Gourdie et al., 1991; Gros and Jongsma, 1996; van Veen et al., 2005b). Within the ventricular wall, Cx43 expression is lower in the epicardium than in deeper regions (Yamada et al., 2004). Unlike Cx43^−/−^ mice, Cx43^+/−^ mice survive and age normally (Betsuyaku et al., 2002), although ventricular and atrial level of Cx43 protein are reduced by approximately 50% (Guerrero et al., 1997; Thomas et al., 1998). There has been a debate about to what extent this reduction in expression affects conduction, with one group reporting a 50% reduction in ventricular conduction velocity (Guerrero et al., 1997; Eloff et al., 2001), and another group reporting no change (Morley et al., 2000). The latter result is in better agreement with studies using mathematical models, which predict a weaker dependence of conduction velocity on gap junctional coupling (Shaw and Rudy, 1997; Wiegerinck et al., 2006) and a study on strands of cultured myocytes from Cx43^+/+^ and Cx43^+/−^ mice, which did not show a difference in conduction velocity (Thomas et al., 2003). Moreover, two later studies in adult mice with conditional deletion of Cx43 showed a reduction of conduction velocity by approximately 40% while Cx43 protein level was decreased by 90% (Gutstein et al., 2001a; van Rijen et al., 2004). Conduction anisotropy and heterogeneity were increased, especially in the RV. VT was readily induced by pacing, often with a stable reentrant circuit in the RV and fibrillatory conduction in the LV (van Rijen et al., 2004). Telemetric recordings revealed that most mice die from arrhythmic sudden death within weeks of the start of Cx elimination (Gutstein et al., 2001a). By contrast, in a conditional knock-out strain with a 50% reduction in Cx43, no change in conduction velocity was observed and no arrhythmias were inducible (van Rijen et al., 2004).

Similar findings were reported in mice with a cardiac-specific deletion with a gradual postnatal decline in Cx43 expression. At birth, cardiac structure in these mice is normal (Eckardt et al., 2006), but Cx43 protein decreases to 59% of control level at 25 days (without a change in conduction velocity) and 18% at 45 days (with a 50% decrease in conduction velocity). At the latter stage, lethal VTs could be induced in 80% of the mice (Danik et al., 2004). Interestingly, optical mapping during sinus rhythm in this model revealed ectopic sites of ventricular activation caused by a paradoxical increase in conduction across Purkinje-ventricular junctions (Morley et al., 2005). In another approach, chimeric mice were produced with a patchy Cx43 expression pattern in the ventricles. In addition to a depressed pump function, these mice showed heterogeneous ventricular conduction and spontaneous non-sustained VT (Gutstein et al., 2001b, 2005).

### Replacement of Cx43

Several studies have assessed whether the role of Cx43 can be taken over by other Cxs, using knock-in mouse models in which Cx43 was replaced by Cx40, 32, 31, or 26. Anatomically, Cx43KI40 hearts showed no abnormalities at birth (apart from mild hypertrophy in some mice), whereas Cx43KI32 hearts showed a mild form of the RV outflow tract abnormalities seen in Cx43^−/−^ mice (Plum et al., 2000). ECG intervals in adult Cx43KI32 and Cx43KI40 mice did not differ from those in control mice. However, spontaneous ventricular extrasystoles were observed more frequently in Cx43KI40 mice than in Cx43KI32 and control mice. In Cx43KI31 mice, the RV outflow tract obstruction is severe, cardiac conduction is markedly slow and mice die within days after birth (Zheng-Fischhofer et al., 2006). In this context it is worth noting that Cx31 is highly restricted in its ability to form channels with other Cxs (Elfgang et al., 1995). Neonatal Cx43KI26 mice do not have structurally abnormal hearts, have only slightly decelerated cardiac conduction, but died within weeks because of deficient lactation of their heterozygous mothers (Winterhager et al., 2007). Adult Cx43KI26 mice (reared by foster mothers) showed a small prolongation in His-to-ventricle conduction time and QRS duration. Thus, several connexins are able to replace Cx43 in the developing and heatlhy adult heart to a large degree. However, the consequences of replacing Cx43 may be more pronounced under pathological conditions (see below for an example in Cx43KI32 mice).

### Response to ischemia

In the ventricles, the closing of gap junction channels in response to ischemia enables the myocardium to “heal-over” (de Mello et al., 1969), but it is also associated with (phase 1b) arrhythmias occurring 15–60 min after the onset of ischemia (De Groot and Coronel, 2004; Wit and Peters, 2012). During acute ischemia, spontaneous and induced VT occurred more frequently in Cx43^+/−^ than in Cx43^+/+^ mice (Lerner et al., 2000). However, infarct size in the weeks after coronary occlusion was smaller in Cx43^+/−^ mice (Kanno et al., 2003). At those later time points, the incidence of spontaneous and induced VT did not differ between Cx43^+/−^ and Cx43^+/+^ mice, suggesting that effect of reduced coupling is offset by the smaller infarct size (Betsuyaku et al., 2004).

Cx43 hemichannels are present in the plasma membrane and can open as a non-selective pore in response to metabolic inhibition, which would accelerate cell death (John et al., 1999; Kondo et al., 2000). Gap 19, a peptide that prevents hemichannel opening, without affecting gap junction channels, reduces ischemia/reperfusion damage (Wang et al., 2013).

In addition, Cx43 is located in the nuclear membrane and in subsarcolemmal mitochondria (Rodriguez-Sinovas et al., 2007; Boengler et al., 2009). Ischemic preconditioning increases the levels of mitochondrial Cx43 (Boengler et al., 2005) and accordingly, ischemic preconditioning is lost in Cx43^+/−^ mice (Schwanke et al., 2002; Li et al., 2004). Cx43 is involved in mitochondrial K^+^ uptake (Miro-Casas et al., 2009) and respiration (Boengler et al., 2012), forming hemichannels in the mitochondrial inner membrane that interact with other proteins (Rodriguez-Sinovas et al., 2007).

The carboxy terminus of the Cx43 protein is an important regulatory domain, involved in e.g., the response to intracellular acidification (Duffy et al., 2004). A mutant with a C terminal truncation shows a redistribution of Cx43 to the periphery of larger, sparser gap junctions (Maass et al., 2007). After ischemia/reperfusion, this mutant has larger infarcts and a higher incidence of induced VT (Maass et al., 2009). Similarly, increased gap junctional coupling by adenoviral transfection of the less pH-sensitive Cx32 increases infarct size in mice with a coronary occlusion (Prestia et al., 2011). Replacement of Cx43 with Cx32 has more complex effects (Rodriguez-Sinovas et al., 2010). Under normal conditions, ATP levels were decreased and lactate levels were increased in the myocardium of these Cx43KI32 mice. Although infarct size following ischemia/reperfusion was smaller, protection by preconditioning was lost, indicating that Cx32 cannot replace Cx43 in its role in mitochondrial metabolism and preconditioning.

### Cx43 mutations

Several models of mutations in Cx genes have been developed that correspond to inherited diseases in humans, as reviewed in (Dobrowolski and Willecke, 2009) and (Delmar and Makita, 2012). The G60S missense mutation in Cx43 acts in a dominant negative way to cause oculodentodigital dysplasia, including various cardiac manifestations (patent foramen ovale, reduced cardiac function, a large decrease in Cx43 due to impaired trafficking to the intercalated disc, a small decrease in ventricular conduction velocity and a variety of ECG abnormalities, both brady- and tachyarrhythmias) (Flenniken et al., 2005; Kalcheva et al., 2007; Manias et al., 2008; Tuomi et al., 2011). The G138R (Dobrowolski et al., 2008) and I130T (Kalcheva et al., 2007) mutants show a similar phenotype of oculodentodigital dysplasia, including similar alterations in cardiac structure and function. However, in the case of G138R, Cx43 trafficking seems to be normal, but Cx43 (hemichannel) function is reduced due to a loss of phosphorylation. The importance of Cx43 phosphorylation is also underscored by a study on mice with “phosphatase resistant” Cx43, in which serine residues 325, 328, and 330 are replaced by either phosphomimetic glutamine (S3E) or non-phosphorylatable alanine (S3A) (Remo et al., 2011). S3E mice were resistant to gap junctional remodeling in response to transverse aortic constriction and showed a lower inducibility of VT than wildtype mice. Conversely, in S3A mice Cx43 was lost from the intercalated disc in response to transverse aortic constriction and inducibility of VT was higher than in wildtype mice.

### Interactions with other proteins

Cx43 interacts with a number of other proteins. Whereas the organizations of adherens junctions is not disrupted in the absence of Cx43 (Gutstein et al., 2003), conversely a loss of E-cadherin, N-cadherin, desmin or desmoplakin does greatly decrease Cx43 in intercalated discs (Ferreira-Cornwell et al., 2002; Gard et al., 2005; Kostetskii et al., 2005; Li et al., 2005; Gomes et al., 2012). Among the many changes in gene expression (directly or indirectly) caused by the absence of Cx43 (Iacobas et al., 2005), potassium current also show regional alterations in Cx43^−/−^ mice, leading to proarrhythmic shortening of the action potential duration (Danik et al., 2008), whereas sodium channel function may (Desplantez et al., 2012; Jansen et al., 2012a) or may not (Johnson et al., 1999) be affected. Short term (6 h) ventricular pacing causes a decrease in Cx43 gene expression (Kontogeorgis et al., 2008a), while the shift in potassium channel expression in response to pacing was altered in Cx43^+/−^ mice (Kontogeorgis et al., 2008b).

Interestingly, inducible deletion of Cx43 leads to increased fibrosis during aging and pressure overload because of enhanced fibroblast activity, with a concomitant increase in conduction heterogeneity and vulnerability to VT (Jansen et al., 2012b). On the other hand, TGFβ—dependent profibrotic signaling in response to myocardial infarction is blunted in Cx43^+/−^ mice compared to Cx43^+/+^ mice, leading to a decrease in post-infarct fibrosis (Zhang et al., 2010b), probably because of the difference in Cx43 expression in fibroblasts (Zhang et al., 2010a). With respect to arrhythmogenesis, these and some other studies highlight the clinically relevant conjunction of changes in excitability, electrical coupling and structural remodeling (van Veen et al., 2005a; Stein et al., 2009) that is poorly captured by monogenic alterations in transgenic mouse models.

## Connexin 45

### Properties and expression

Cx45 forms channels with small conductance of 30 pS that are the most sensitive to transjunctional voltage of all cardiac Cxs (V_1/2_ = 13 mV, *G*_*j*, min_ = 0.12) (Moreno et al., 1995). Cx45 is the connexin expressed earliest in the developing heart, and the only one present before embryonic day 9. Cx45-deficient mice develop conduction block and a cushion defect caused by impairment of the epithelial-mesenchymal transformation of the cardiac endothelium (Kumai et al., 2000). Mice with cardiac myocyte-specific deletion of Cx45 do not have the cushion defect, but still develop conduction block and also die at around embryonic day 10 from pump failure (Nishii et al., 2003). The function of Cx45 in early cardiac development cannot be replaced by the neuronal connexin Cx36 (Frank et al., 2010).

### Pacemaker and conduction system

During later embryonic development, Cx45 becomes increasingly localized to pacemaker and conduction system Alcolea et al., 1999; Coppen et al., 1999. In adult mice, Cx45 is the main connexin in the central SA node, a region apposed by protrusions of Cx40 and 43 positive atrial cells (Verheijck et al., 2001). Nevertheless, the heart rate is unaffected in mice with a heart-specific inducible Cx45 deletion (Frank et al., 2012). Furthermore, Cx45 is expressed along the entire AV conduction system, including the AV node, His bundle, proximal bundle branches and Purkinje system (Coppen et al., 1999; van Veen et al., 2005b). In adult mice, the dependence of AV conduction on the 3 Cxs expressed in the AV node region is complex. As noted above, mice deficient in Cx30.2 and 40 show accelerated and decelerated AV conduction, respectively (Schrickel et al., 2009). Apparently, Cx45 is sufficient for AV conduction, because mice deficient in both Cx30.2 and 40 show normal AV conduction (Schrickel et al., 2009). In mice with a heart-specific, inducible deficiency in Cx45, AV nodal conduction is slower than normal (Frank et al., 2012), whereas atrial and ventricular conduction was not affected (Bao et al., 2011). Interestingly, these mice also displayed a posttranslational reduction of Cx30.2 expression in the conduction system. Combining the inducible deficiency in Cx45 with a deficiency in Cx30.2 further slowed AV nodal conduction (Frank et al., 2012). Although a heterozygous deletion of Cx45 does not prolong the PR interval by itself, it does further increase the observed PR prolongation in Cx40-deficient mice (Kruger et al., 2006).

### Working myocardium

Cx45 was initially thought to be widely expressed in the mammalian working myocardium (Davis et al., 1994; Verheule et al., 1997). Coppen et al later showed that this observation was mainly caused by cross-reactivity of a Cx45 antibody with Cx43 (Coppen et al., 1998). Low levels of Cx45 are expressed in the mouse ventricular working myocardium, amounting to 0.3% of total gap junction protein (Bao et al., 2011). Cx45 can form both heterotypic (Elenes et al., 2001) and heteromeric (Desplantez et al., 2004) channels with Cx43. However, co-expression of Cx45 with Cx43 appears to decrease the size of gap junctions (Grikscheit et al., 2008). This phenomenon could be more important under conditions with a higher Cx45/Cx43 ratio, for example in the ventricular epicardium, where Cx43 levels are relatively low (Yamada et al., 2004), or during heart failure, where the expression of Cx45 increases and Cx43 decreases (Yamada et al., 2003). Indeed, mice overexpressing Cx45 in the heart showed a reduction in gap junctional coupling and an increased inducibility of VT (Betsuyaku et al., 2006).

## Connexin 46

Along with Cx50, Cx46 is the main connexin expressed in the lens, forming channels with a γ*j* = 140 pS in CsCl, V_1/2_ = 48 mV, *G*_*j*, min_ = 0.11 (Hopperstad et al., 2000). mRNA for Cx46 (and Cx50) has been detected in the adult canine heart (Davis et al., 1994). The localization of Cx46 (and Cx50) protein in the dog heart is unknown, although one study detected minimal Cx46 immunoreactivity between some atrial and ventricular myocytes (Davis et al., 1995). However, in neonatal mice Cx46-positive myocytes were detected in the cardiac conduction system (or more precisely in the atrium, AV canal, intraventricular septum and ventricular subendocardium) (Chi et al., 2010). Cx46-deficient mice displayed a slightly lower heart rate, a prolonged QRS and QT duration and a QRS morphology consistent with bundle branch block (Chi et al., 2010). Cx46 has a propensity to form functional hemichannels (Trexler et al., 1996; Pfahnl and Dahl, 1999), but their role in the heart is unknown.

## Conclusions

The diversity of connexins in the heart allows fine-tuning of electrical coupling depending on the region and on conditions. As therapeutic strategies, both increasing and decreasing gap junctional coupling could in theory be beneficial under certain circumstances. However, a non-specific treatment targeting gap junctions may have beneficial effects in one part of the heart but cause deleterious side effects in another. Several compounds have been developed that can modulate gap junctions (Herve and Dhein, 2010; De Vuyst et al., 2011). Some of these have been successfully tested in animal models. For example, in canine model of heart failure, enhancing gap junctional coupling with rotigaptide can reduce the vulnerability to atrial fibrillation (Guerra et al., 2006). For more selective pharmacological manipulation of gap junction coupling, it is desirable for a treatment to be (1) specific to the heart and/or (2) specific for a particular connexin. In this respect, gap junction blocking “peptidomimetics,” a class of small peptide molecules that have some Cx selectivity, are especially promising (Evans et al., 2012). An enormous amount of knowledge on cardiac connexins has been gathered from genetically engineered mouse models, as described above. There are some important differences in cardiac electrophysiology in general and connexin distribution in particular between murine and human hearts (Kaese and Verheule, 2012). Nevertheless, for the development of connexin-specific treatment strategies, knowledge derived from transgenic mouse models provides a wealth of valuable insights.

### Conflict of interest statement

The authors declare that the research was conducted in the absence of any commercial or financial relationships that could be construed as a potential conflict of interest.
